# Type II toxin: antitoxin systems. More than small selfish entities?

**DOI:** 10.1007/s00294-015-0541-7

**Published:** 2015-11-23

**Authors:** Andrea Rocker, Anton Meinhart

**Affiliations:** Department of Biomolecular Mechanisms, Max Planck Institute for Medical Research, Jahnstrasse 29, 69120 Heidelberg, Germany

**Keywords:** Toxin–antitoxin system, Fusion, Multi-domain protein, Elongated toxin

## Abstract

Toxin–antitoxin (TA) modules regulate metabolism and viability of bacteria and archaea. In type II TA systems these functions are generally thought to be performed by two small proteins. However, evidence is increasing that the toxins are much more diverse and can form multi-domain proteins. Recently, we published a novel type II TA system in which toxin and antitoxin are covalently linked into a single polypeptide chain. In this review we summarize the current knowledge on these elongated toxin homologs and provide perspectives for future study.

Bacterial type II toxin–antitoxin (TA) systems were already identified in 1983 as bicistronic operons that ensure stable plasmid maintenance in bacteria (Ogura and Hiraga 1983). They are generally thought to consist of a small harmful enzyme (toxin) and a small proteinaceous antidote (antitoxin) (Goeders and Van Melderen 2014; Yamaguchi et al. 2011). Upon binding to each other, a neutral protein–protein TA-complex is formed under non-stress condition that remains inactive in the cytoplasm of the producing bacterial organism. Cellular concentrations of type II TA complexes are often regulated by autoregulation of transcription by the antitoxin or toxin–antitoxin complex (Gerdes and Maisonneuve 2012) and simple repression by promoter binding is in some cases further enhanced by conditional cooperativity (Garcia-Pino et al. 2010). Activation of the toxin is accomplished by degradation of the antitoxin by cellular proteases liberating the proteolytically stable toxin (Schuster and Bertram 2013). By this simple mechanism, plasmid encoded type II TA systems lead to post-segregational cell death in bacterial progeny that are devoid of the plasmid. Although originally thought to mainly serve by this mechanism as addiction modules that ensure the maintenance of bacterial populations containing stably inherited plasmids (Engelberg-Kulka and Glaser 1999), type II TA systems are also now considered as important modulators of bacterial physiology for instance as stress response loci (Gerdes et al. 2005, Wang and Wood 2011). Moreover, type II TA systems are highly prevalent on the chromosome of bacteria and additional functions apart from being simple selfish entities that secure stable maintenance of mobile genetic elements are reported (Van Melderen and Saavedra De Bast 2009). In fact, four additional different roles have been recently been attributed to type II TA systems which are still a matter of debate in some cases (Van Melderen 2010); these functions range from being modules that induce programmed-cell death activated by a variety of unrelated stressful conditions where altruistic death provide nutriments for the remaining bacteria as reported for the chromosomal encoded *mazEF* locus from *Escherichia coli* (Engelberg-Kulka et al. 2006), modules that lead to global inhibition of translation as it is the case for the *relBE* locus from *E. coli* (Christensen et al. 2001), modules such as the *hipAB* from *E. coli* locus that induces persistence of a small fraction of bacteria (Correia et al. 2006, Korch et al. 2003) upon phosphorylation of the glutamyl-tRNA synthetase (Germain et al. 2013; Kaspy et al. 2013) to developmental regulators as reported for the *mazEF* locus from *Myxococcus xanthus* (Nariya and Inouye 2008). Additionally, other type II TA systems have been shown to influence biofilm formation (Kim et al. 2009) or virulence (Harvey et al. 2011). Noteworthy, in addition to the here exemplified type II TA systems, similar functions have been ascribed for a number of other type II TA systems as well, and even overlapping functions have been proposed for some systems (De la Cruz et al. 2013; Magnuson 2007; Van Melderen and Saavedra De Bast 2009; Wang and Wood 2011). Similar as type II TA systems are diverse in their function their cognate toxins also interfere with a number of important cellular processes. Type II TA systems interfere for instance with replication and transcription by inhibiting Topoisomerases (Bernard and Couturier 1992; Harms et al. 2015; Jiang et al. 2002; Maki et al. 1992) or impair replication through their interaction with the beta Sliding clamp of DNA polymerase III (Aakre et al. 2013). Interestingly, a huge number of type II TA-systems interfere with translation by either acting as RNA interferases or directly inhibiting the ribosome (Castro-Roa et al. 2013; Guglielmini and Van Melderen 2011). More recently, type II TA-systems were also shown to impair with cell wall synthesis (Mutschler et al. 2011) or cell division (Masuda et al. 2012), thereby evoking cell death or stasis. Bioinformatic analyses browsing the increasing number of available microbial genomes revealed that type II TA systems are widespread in both bacteria and archaea where they can exist in multiple copies on plasmids as well as on chromosomes (Leplae et al. 2011; Makarova et al. 2009; Sberro et al. 2013; Sevin and Barloy-Hubler 2007; Shao et al. 2011). Hitherto, type II TA systems have been grouped into 12 different families, depending on the toxin primary sequence and activity (Leplae et al. 2011). Nevertheless, the current picture on type II TA system seems to be far from complete.

The commonly used bioinformatic approach for de novo identification of type II TA system is based on the occurrence of bicistronic operons, of which the second translational product encodes a putative toxic enzyme (Leplae et al. 2011; Sberro et al. 2013; Shao et al. 2011). Homology searches have greatly advanced our knowledge of those systems and even distantly related type II TA systems have been identified. However, a more complex search for type II TA systems revealed a number of unusually long and sometimes even solitary toxin genes in the genome of different bacteria (Makarova et al. 2009; Sevin and Barloy-Hubler 2007). The cellular function of these novel and remarkable type II TA loci is poorly characterized and most of these systems are scientifically orphaned, so far.

One of the first examples of such long type II toxins is encoded by the *phoH2* locus found in the genomes of *Mycobacterium tuberculosis* and *Mycobacterium smegmatis*, but also in the thermophilic organism *Thermobispora bispora* (Andrews and Arcus 2015). In contrast to all previously reported type II toxins that are small, single-domain proteins, the toxin PhoH2 was shown to be a two-domain protein consisting of an N-terminal VapC toxin-like PIN domain linked to a C-terminal PhoH helicase domain adding up to a total molecular weight of approximately 50 kDa. The latter ATPase domain was shown to have RNA binding and unwinding activity, complementing the RNase activity of the N-terminal VapC toxin domain. PhoH2 is co-transcribed from a bicistronic operon together with the open reading frame of the PhoAT antitoxin that binds to PhoH2. Finally, expression of *M. tuberculosis* PhoH2 in *M. smegmatis* was reported to have a negative effect on growth, which was alleviated by the presence of the adjacently encoded PhoAT antitoxin. The authors speculate that the acquisition of additional domains expands the versatility of the mycobacterial VapBC TA systems, which function as posttranscriptional regulators in response to environmental stress (Andrews and Arcus 2015).

Another variety of unconventional and previously unrecognized type II TA system is the multi-domain and auto-regulated EzeT system encoded by a variety of different strains from *Escherichia coli* and several related organisms (Rocker and Meinhart 2015). We have shown that the C-terminal domain of this type II TA system harbors a zeta-toxin like UDP-*N*-acetylglucosamine kinase domain that interferes with peptidoglycan synthesis similar to the related toxins from *Streptococcous pneumoniae* and *Streptococcus pyogenes* (Mutschler et al. 2011; Tabone et al. 2014). In contrast to conventional type II TA systems, EzeT contains an N-terminal antitoxin domain that is fused with the kinase domain (Rocker and Meinhart 2015). This covalent linkage of toxin and antitoxin functionalities within a single polypeptide chain leads to an auto-regulated toxin which had most likely evolved by fusion of a conventional bicistronic operon. The action of an antitoxin *in cis* abolishes passive complex diffusion and therefore provides additional possibilities for regulation. Activation could be either achieved by protease cleavage, partial digestion, chaperone binding or protein modification. Moreover, the generally observed recovery of a toxin-poisoned bacterial population by newly synthesized antitoxin until the “point of no return” is not feasible in such systems, as toxin and antitoxin are synthesized in equimolar amounts and are additionally fused. Interestingly, a similar fusion protein TA system has been reported for the Phd antitoxin and a RelE toxin, yb_293189 TA system from *Ralstonia eutropha* (Loris and Garcia-Pino 2014). However, in this case truncations are thought to abolish enzymatic activity of the toxin as well as the inhibitory function of the antitoxin. Whether this system is a dead-end product in evolution or constitutes a functional system awaits further investigations.

Furthermore, additional evidence for an expansion of toxin functions is provided by domain analyses in the protein family database PFAM (Finn et al. 2014). For example, multi-domain zeta toxin homologs (PF06414) are predicted but so far uncharacterized. In this family members are annotated in which the zeta core regions of roughly 200 amino acids are predicted to be linked to phosphatase or peptidoglycan binding domains. In addition, zeta homologs with more than 500 amino acids have been reported previously (Chan et al. 2012). However, no experimental studies of these systems have been performed, yet. Similarly, ParE-like toxins (PF05016), Fic/Doc-domains (PF02661) and PemK family toxins (PF02452) have been described as parts of multi-domain proteins. In addition, 200 amino acid long proteins combining a 60 amino acid long N-terminal region with homology to the toxin HicA (PF07927) with a 130 amino acid long region homologous to the HicB-like antitoxin (PF15919) as well as an HicB domain (PF05534) are assigned, indicating a putative TA fusion protein.

It seems that investigations of elongated toxin proteins still hold unanticipated number of surprising findings as many novel functionalities and regulatory concepts can be acquired by domain fusion, adding to the complexity of TA systems.
